# Is stress related to itch in German students? Results of an online survey

**DOI:** 10.3389/fmed.2023.1104110

**Published:** 2023-04-25

**Authors:** Stephanie Kiupel, Jörg Kupfer, Sophia Kottlors, Uwe Gieler, Gil Yosipovitch, Christina Schut

**Affiliations:** ^1^Institute of Medical Psychology, Faculty of Medicine, Justus Liebig University, Giessen, Germany; ^2^Department of Dermatology and Allergology, Justus-Liebig-University, Giessen, Germany; ^3^Clinic for Psychosomatics, Vitos Klinik, Giessen, Germany; ^4^Department of Dermatology and Cutaneous Surgery and Itch Center, Miller School of Medicine, University of Miami, Miami, FL, United States

**Keywords:** pruritus, skin symptoms, perceived stress, students, self report

## Abstract

**Introduction:**

German students report to be more stressed than the general population. Highly stressed students from other countries (United States, Australia, Saudi-Arabia) were found to have more skin symptoms, including itch, than lowly stressed students. The current study aimed to assess whether itch is associated with stress in a larger sample of German students.

**Methods:**

838 students (3.2% of all invited students) took part in the questionnaire based study and filled in the Perceived Stress Questionnaire as well as a modified version of the Self-Reported Skin Questionnaire. Students were categorized into highly (HSS) and lowly stressed students (LSS) by determination of the 25th and 75th percentile.

**Results:**

Itch occurred significantly more often in HSS compared to LSS (OR = 3.41 (2.17–5.35)). In addition, itch intensity was significantly related to perceived stress.

**Discussion:**

These findings not only highlight the importance of offering stress management trainings also to students in Germany in order to minimize itch, but also encourage future research on stress and itch in certain student subgroups.

## Background

Studying often implies having to cope with a variety of stressors. One year after having started university, students report a decline in physical and psychological well-being, especially due to high study demands, difficult time management or low social support (1). Moreover, students name relationship stressors, trying to fulfill expectations from self and others or lack of resources (e.g., lack of time, money or support) as stressors they have to face (2).

Perceived stress differs in students from different countries. In one study, a greater proportion of medical students from a Middle Eastern country (not named) reported stress compared to US medical students (75% vs. 58%) (3). Eventhough in Germany studying is less expensive than in other countries like the US, German students (in this case first year medical students) still report high amounts of stress compared to a reference sample of the general population (4). Studies showed that 21–36% of German students suffer from high or very high levels of stress (5, 6). 25% of the students feel clearly overstrained (6). Kötter and Voltmer (7) assessed medical students’ stress levels and their physical and psychological health status. The study showed that highly stressed students in comparison to lowly stressed students had significantly worse physical and psychological health, characterized, e.g., by lower physical or social functioning.

Thus, an association between health and stress has been shown for students. Regarding dermatological conditions, relationships between stress and the intensity of various skin diseases have already been reported (8–10). An association between stress and itch has been shown in patients with skin diseases like psoriasis (11, 12) and atopic dermatitis (13, 14), but also in the general population (15).

So far, three studies, conducted in the US (16), Australia (17) and Saudi Arabia (18), investigated the relationship between stress and skin symptoms in student populations. The Saudi-Arabian study (18) included medical students only. These studies found that students with high perceived stress levels reported to have itch significantly more often than students with low perceived stress levels.

The present study aims to replicate these findings in a sample of German students. We hypothesize that also in German students we will find a positive relationship between reported stress levels and the occurrence of itch. A second aim of the study is to investigate whether stress is also related to other skin symptoms in German students.

## Materials and methods

All persons studying at the Justus-Liebig University Gießen in the summer semester of 2015 (*n* = 26,060) were made aware of this questionnaire study *via* three circular emails. The first email was sent around in mid May, while the second and third emails were sent around three and 6 days later, respectively. Thus, data collection was finished within 7 days. Students were instructed to only participate once. They were informed that the aim of this study was to investigate the relationship between stress and skin symptoms in German students and further that it would take approximately 10 min to participate. Moreover, they were told that they had the chance to win one of three vouchers after participation. Participation in the raffle was voluntary. The email contained a link to the questionnaire.

Of all contacted students, 838 (3,2%) agreed to participate. 44 students were excluded due to incomplete responses, unspecified gender or being younger than 18 or older than 30 years. Electronic informed consent was obtained from all participants. Before the beginning of the study it was approved by the Ethics committee of the Medical Faculty of the Justus-Liebig-University Giessen.

### Measures

#### Stress

To assess the students’ perceived stress levels we used the German version of the Perceived Stress Questionnaire (PSQ) (19). The PSQ consists of 30 items, which need to be answered on a scale from 1–4 (1: almost never, 2: sometimes, 3: often, and 4: usually). This instrument measures self-reported psychological stress within the last 4 weeks. It comprises seven subscales, namely harassment (example item: “You are under pressure from other people“), overload (“You have too many things to do“), irritability (“You are irritable or grouchy“), lack of joy (“You feel lonely or isolated“), fatigue (example item: “You feel tired“), worries (example item: “You are afraid for the future “) and tension (example item: “You have trouble relaxing”). A PSQ-raw score can be calculated by inverting the items 1,7,10,13,17,21,25,29 and summing up the values for all 30 items afterwards. This raw score is then inserted in the formula: (raw score – 30)/90 in order to receive the PSQ index. This can range from 0–1 with 0 indicating no stress and 1 indicating highest levels of stress. In the validation sample, persons scoring ≤0.3 fell into the lowest quartile, while persons with scores ≥0.52 fell into the highest quartile (19). We decided not to use these cut-off-values, but to calculate the 25th and 75th percentile for our sample instead, because this approach has also been chosen in the former US-, Australian and Saudi-Arabian studies. In our sample, students scoring ≤0.3 were classified as lowly stressed students and students scoring ≥0.57 were regarded as highly stressed students.

#### Itch and other skin symptoms

To assess itch and other skin symptoms, we applied a modified version of the Self-Reported Skin Questionnaire (SRSQ) (20). The SRSQ measures the occurrence and extent of different skin symptoms during the last 7 days. In this study we extended this time period to 4 weeks in order to align it with the time period of the PSQ. The SRSQ items were answered on a 4-point scale with 1 representing “no complaints,” 2 “a little,” 3 “quite a lot” and 4 “very much.” For further analyses we dichotomized these answers as to whether itch and other skin complaints did or did not occur in the students. This gave us the opportunity of comparing students stating they had “no complaints” to students who fell into the other three answer categories.

#### Itch intensity

In addition, we asked the students to rate their itch intensity during the last 4 weeks on a Visual Analog Scale (VAS), ranging from 0 (“no itch at all”) to 10 (“worst itch ever”).

#### Statistics

Data analyses were conducted using SPSS 24. For the main analysis participants were grouped into lowly stressed students (LSS) and highly stressed students (HSS) by determination of the 25th and 75th percentile of the PSQ index. Students in between, who were neither categorized as lowly nor highly stressed, were regarded as moderately stressed. These were not included in the analysis. In order to compare LSS and HSS regarding sociodemographic variables and itch intensity we computed t-tests for independent groups in case of continuous variables and chi square-tests in case of nominal variables. Odds ratios and 95% confidence intervals were calculated in order to investigate whether skin symptoms, including itch, were more prevalent in HSS than in LSS. For further analysis of the relationship between itch and stress we determined the 25th and 75th percentile for all PSQ-subscales separately as well. Again, we calculated odds ratios and 95% confidence intervals.

## Results

### Sample characteristics

The final sample size comprised *n* = 794 students, of whom 659 (83%) were female. The mean age ± SD of the subjects was 23.1 ± 2.7 years. Of the total sample, 207 students were classified as LSS and 201 as HSS (one person with non-specified gender was excluded from this group). Gender distribution and age did not differ between HSS and LSS (*p* > 0.05).

### Relationship between stress and itch

HSS significantly more often reported to have itch compared to LSS (*p* < 0.001, OR 3.41 (95% CI 2.17–5.35)). In addition, itch intensity in HSS was significantly higher than in LSS (*p* < 0.05; 3.02 ± 2.46 vs. 1.51 ± 1.79). Further analyses revealed that students with high scores on the PSQ-subscales more often reported itch than students with low scores on the subscales (all *p* < 0.05). The results are shown in Table 1.

**Table 1:** Percentage of persons who reported itch in the group of highly stressed students (HSS) and lowly stressed students (LSS).

Scale that was used to determine whether a person was highly or lowly stressed	% of HSS reporting itch	% of LSS reporting itch	OR (95 % CI)
PSQ-total score	81.6 %	56.5 %	3.41 (2.17–5.35)
Harrassment (S1)	76.9 %	56.3 %	2.57 (1.71–2.93)
Overload (S2)	74.8 %	63.3 %	1.73 (1.20–2.48)
Irritability (S3)	78.3 %	61.6 %	2.25 (1.52–3.33)
Lack of joy (S4)	81.8 %	56.8 %	3.41 (2.02–5.77)
Fatigue (S5)	80.4 %	55.4 %	3.31 (2.21–4.97)
Worries (S6)	80.4 %	59 %	2.85 (1.85–4.39)
Tension (S7)	78.2 %	56.9 %	2.72 (1.83–4.04)

OR, odds ratios; CI, confidence interval; S, subscale. Participants were grouped into LSS and HSS by determination of the 25th and 75th percentiles of the total PSQ-index as well as by determination of the 25th and 75th percentiles of the seven PSQ-subscales (S1-7).The third column represents corresponding OR for HSS vs LSS (95% confidence interval).

### Relationship between stress and skin complaints

HSS reported oily, waxy or flaky patches on the scalp, scaly skin, nail-biting (onychophagia), itchy rashes on hands, hair pulling (trichotillomania), other rashes on face, dry/sore rashes, pimples and warts more often than LSS (all *p* < 0.05). For more details see Table 2.

**Table 2:** Percentage of students who reported to have specific skin symptoms in the group of highly stressed students (HSS) and lowly stressed students (LSS).

Skin symptoms	% of HSS reporting to have specific symptoms	% of LSS reporting to have specific symptoms	OR (95 % CI)
Flaky patches on the scalp	54.2 %	30.0 %	2.90 (1.93 – 4.37)
Scaly skin	65.7 %	44.4 %	2.39 (1.60 – 3.57)
Nail-biting (Onychophagia)	36.8 %	26.6 %	1.61 (1.06 – 2.45)
Itchy rash on hands	38.3 %	15.9 %	3.27 (2.05 – 5.23)
Hair pulling (trichotillomania)	11.4 %	3.9 %	3.21 (1.40 – 7.37)
Other rashes on face	28.4 %	11.1 %	3.17 (1.86 – 5.39)
Dry/sore rash	43.3 %	26.1 %	2.16 (1.43 – 3.28)
Pimples	85.6 %	77.3 %	1.74 (1.05 – 5.56)
Warts	15.9 %	6.3 %	2.83 (1.44 – 5.56)

OR, odds ratios; CI, confidence interval. Students were regarded as HSS in case they had a PSQ total score falling into the highest quartile of the sample; students were regarded as LSS in case they had a PSQ total score falling into the lowest quartile of the sample. Illustrated are also corresponding OR for HSS vs LSS in PSQ total score (95% confidence interval).

## Discussion

The aim of the present study was to analyze whether self-rated stress and itch are related in a sample of German university students. The study revealed that HSS more often reported to have itch compared to LSS. It is striking that in this study, the prevalence of itch was very high with 81.6% in HSS and 56.5% in LSS. Other studies found a prevalence of 8.4% or 25.4% in the general population (21, 22). The major difference between these studies and the current study can possibly be explained by the different time intervals for which itch was assessed. In our study, we asked students whether they had itch during the last month, while the time intervals in the other studies were one week (21) or current moment (22).

Similar results were noted for the PSQ-subscales: Students with high scores regarding harassment, overload, irritability, lack of joy, fatigue, worries and tension more often had itch than students with low scores on these subscales. In addition, itch intensity, measured *via* VAS, was related to self-rated stress with significantly higher scores in the group of HSS compared to LSS.

Our results regarding the relationship between stress and itch are not only in line with the results of previous studies from the United States (16), Australia (17) and Saudi Arabia (18), but also with several former investigations which found relationships between stress and itch in people with skin diseases (e.g. 8) and the general population (15). Besides the connection between stress and itch, we also found that HSS compared to LSS more often reported a variety of other skin complaints of which the majority is itchy, such as oily, waxy or flaky patches on the scalp. These symptoms are suggestive of seborrheic dermatitis that is often itchy and associated with stress (23). Also scaly skin, itchy rashes on hands, hair pulling, other rashes on face and dry/ sore rashes were associated with stress. These findings are similar to reported associations between stress and the occurrence of different skin symptoms (24–27). Furthermore, it is important to note, that an exacerbation of itch can further worsen stress, leading to a vicious cycle of itching and scratching that significantly impairs patients’ quality of life (28).

There are some limitations that need to be mentioned. A limitation of this study is the low response rate of only 3.2% which occurred eventhough many efforts (e.g., raffle of vouchers, short duration of the questionnaire) were made to increase it. A second limitation lies in the gender distribution as 83% of the participants were female. Thus, future studies should especially try to recruit non-female persons in order to receive a better picture of the relationship between stress and skin symptoms in males and persons with non-binary gender also. Moreover there may be some selection biases as it is possible that those students who pay more attention to their skin due to more itch and skin complaints as well as more students with high amounts of perceived stress particularly agreed to participate in the online survey. Another shortcoming is the time period to which the itch assessment as well as the assessment of the other skin symptoms refers. As we asked about the occurrence of itch and itch intensity within the last 4 weeks, we cannot control for a possible memory bias. It is possible that the reported itch differed from the itch that actually occurred during that time period. In line with this thought, a review (29) about memory for pain revealed quite an inconsistent picture regarding the comparison of patients’ actual pain sensation and their reports on remembered pain sensation afterwards.

Nevertheless, our findings encourage the implementation of interventions that aim to lower students’ stress levels and through that possibly the occurrence of itch and other skin symptoms. Previous studies demonstrated benefits of psychological interventions in people suffering from itch (30, 31) or the itchy skin disease psoriasis (32). Furthermore, studies investigating stress reducing interventions in university students particularly found reduced anxiety and psychological distress (33–35), mood disturbances (36) or better emotional adjustment (37) among students.

An interesting next step could be to examine whether a relationship between itch and stress occurs more often in certain study disciplines. According to a study by a German insurance company (38), veterinary medicine, agricultural sciences, nutritional sciences and computer sciences students seem to exhibit the highest stress levels, while students studying cultural sciences, linguistic, arts, teaching and sport sciences display the lowest stress levels. Future studies should also compare the relationship between students’ stress levels and their skin symptoms in different countries as the relationships may differ in different environments and cultures.

## Data availability statement

Data will be made available by the corresponding author upon reasonable request.

## Ethics statement

The studies involving human participants were reviewed and approved by Ethics committee of the Medical Faculty of the Justus-Liebig-University Giessen. The ethics committee waived the requirement of written informed consent for participation.

## Author contributions

StK: formal analysis (lead), investigation (equal), methodology (equal), project administration (support), visualization (lead), and writing – original draft (equal). JK: conceptualization (support), investigation (equal), methodology (support), project administration (support), and writing – review and editing (equal). SoK: conceptualization (support), investigation (lead), data acquisition (lead), and writing – review and editing (equal). UG and GY: conceptualization (support), methodology (support), and writing – review and editing (equal). CS: conceptualization (lead), formal analysis (support), methodology (equal), project administration (lead), supervision (lead), visualization (support), and writing – original draft (equal). All authors contributed to the article and approved the submitted version.

## Conflict of interest

The authors declare that the research was conducted in the absence of any commercial or financial relationships that could be construed as a potential conflict of interest.

## Publisher’s note

All claims expressed in this article are solely those of the authors and do not necessarily represent those of their affiliated organizations, or those of the publisher, the editors and the reviewers. Any product that may be evaluated in this article, or claim that may be made by its manufacturer, is not guaranteed or endorsed by the publisher.
